# Health-related quality of life up to 2 years after SARS-CoV-2 infection: a descriptive cohort study

**DOI:** 10.1017/S0950268825000366

**Published:** 2025-03-31

**Authors:** Elias A. M. Abucar, Mascha Kern, Tobias Kurth, Anne Meierkord, Maximilian Gertler, Joachim Seybold, Stefanie Theuring, Frank P. Mockenhaupt

**Affiliations:** 1 Institute of International Health, Charité Center for Global Health, Charité – Universitätsmedizin Berlin, Corporate Member of Freie Universität Berlin and Humboldt-Universität zu Berlin, Berlin, Germany; 2 Institute of Public Health, Charité – Universitätsmedizin Berlin, Corporate Member of Freie Universität Berlin and Humboldt-Universität zu Berlin, Berlin, Germany; 3 Centre for International Health Protection, Robert Koch Institute, Berlin, Germany; 4 Medical Directorate, Charité – Universitätsmedizin Berlin, Corporate Member of Freie Universität Berlin and Humboldt-Universität zu Berlin, Berlin, Germany

**Keywords:** COVID-19, SARS-CoV-2, Health-related quality of life, Patient Reported Outcomes

## Abstract

Health-related quality of life (HRQoL) in the context of COVID-19 is not fully understood. We assessed HRQoL using Patient-Reported Outcomes Measurement Information System® measures among 559 former COVID-19 patients and 298 non-infected individuals. HRQoL was captured once up to 2 years after the initial test. Additionally, we described associations of characteristics with impaired HRQoL. Overall, HRQoL scores were inferior among former patients. A meaningful group difference of at least three T-score points was discernible until 12 months after testing for fatigue (3.1), sleep disturbance (3.5), and dyspnoea (3.7). Cognitive function demonstrated such difference even at >18 months post-infection (3.3). Following dichotomization, pronounced differences in impaired HRQoL were observed in physical (19.2% of former patients, 7.3% of non-infected) and cognitive function (37.6% of former patients, 16.5% of non-infected). Domains most commonly affected among former patients were depression (34.9%), fatigue (37.4%), and cognitive function. Factors that associated with HRQoL impairments among former patients included age (OR ≤2.1), lower education (OR ≤5.3), and COVID-19-related hospitalization (OR ≤4.7), among others. These data underline the need for continued attention of the scientific community to further investigate potential long-term health limitations after COVID-19 to ultimately establish adequate screening and management options for those affected.

## Introduction

Ongoing symptoms after Coronavirus Disease 2019 (COVID-19) affect a substantial proportion of individuals following Severe Acute Respiratory Syndrome Coronavirus Type 2 (SARS-CoV-2) infection [1–3]. The majority of individuals affected by those long-term health limitations experience reduced health-related quality of life (HRQoL) [4]. Even though HRQoL reductions after COVID-19 are, therefore, presumably very common in the entire population, more than 5 years after the emergence of SARS-CoV-2, standardized screening and management options of HRQoL limitations after COVID-19 have not been established.

Impairment of the individual’s well-being and/or functioning may be measured as HRQoL, including physical-, emotional-, and social health aspects [5]. Multiple tools have been established to measure and compare HRQoL and have widely been used in clinical and research settings. HRQoL limitations after SARS-CoV-2 infection have been associated with demographic features such as female sex and with characteristics of the acute COVID-19 manifestation, such as hospitalization and mechanical ventilation, among others [6]. However, especially in the predominant group of patients with mild COVID-19, the magnitude, duration, and details of impaired HRQoL are only poorly understood. Similarly, socio-demographic and socio-economic characteristics are known to influence the risk and course of SARS-CoV-2 infection, but their role in post-COVID HRQoL has been investigated only in a limited number of studies [7–10].

Charité – Universitätsmedizin Berlin opened a public SARS-CoV-2 testing site immediately after the first COVID-19 patient had been identified in the city on 1 March 2020. The site provided polymerase chain reaction (PCR)-based testing for the general population with indicative symptoms or contact with SARS-CoV-2-infected individuals and was operative until 18 June 2021 [11, 12]. We followed up positively and negatively tested individuals at a single time point 6 to 26 months after their SARS-CoV-2 test at the Charité public testing site. The primary aim of this study was to evaluate HRQoL among former COVID-19 patients as well as a group of consistently non-infected individuals. The secondary aim was to aid the formulation of hypotheses about potential at-risk groups by exploratively describing associations between socio-demographic and socio-economic factors with impaired HRQoL among formerly SARS-CoV-2-infected individuals.

## Methods

### Study design, setting, and participants

We conducted a matched cohort study among adult participants who had been PCR-tested for SARS-CoV-2 through the public testing site of Charité – Universitätsmedizin Berlin between March 2020 and June 2021. Substantial parts of the methods have also been described by Meierkord *et al.* [12]. Between December 2021 and March 2022, we distributed study invitations, study information, consent forms, and paper-based questionnaires among all individuals who had tested positive for SARS-CoV-2 by postal mail. Documents were in German but upon request, English, Turkish, and Polish versions were re-sent. Responding former COVID-19 patients were included in our cohort. For each former COVID-19 patient, we then identified individuals who had tested negative for SARS-CoV-2 and matched by test date (test week ±2 weeks), age decade, and sex. These individuals were equally invited for study participation by sending them the same documents and included in the cohort if responding. Matching was performed to better standardize the factors mentioned above, not to remove confounding. Former COVID-19 patients completed the questionnaire between December 2021 and June 2022, whereas negatively tested participants did so between January and June 2022. Hereafter, the time of questionnaire completion, i.e. the time of HRQoL assessment, is referred to by ‘study participation’. Individuals who tested negative at presentation to the Charité testing site but reported a confirmed or highly suspected SARS-CoV-2 infection previously or subsequently were excluded. Confirmed or highly suspected COVID-19 was defined by a self-reported positive SARS-CoV-2 test (PCR, antigen, or antibody), common symptoms for multiple days including anosmia and/or ageusia and/or common symptoms after contact with a confirmed COVID-19 patient. Consequently, we assumed that the group of negatively tested individuals had never contracted COVID-19 at any point before study participation. Among participants who send back a completed questionnaire, three tablets were raffled. [12] We categorized former COVID-19 patients into three groups based on the time interval between the initial positive test result and study participation: i1 (≤12 months), i2 (>12–18 months), and i3 (>18 months). Those who did not provide a date of questionnaire completion could not be grouped into i1-3 and are not included in respective parts of the analysis.

### Variables and HRQoL assessment

The study questionnaires included items to assess socio-demographic and socio-economic characteristics and information on previous COVID-19. We defined ‘lower education’ by primary or secondary education, without university qualification, or the absence of formal education. We defined ‘higher non-academic education’ by completed apprenticeship and ‘higher academic education’ when participants had obtained at least university qualification. Derived from the definition of the German Federal Statistical Office we defined ‘family history of migration’ (hereafter, ‘migration background’) when at least one parent was not born in Germany [13]. Since 37% of Berlin’s inhabitants have a migration background, and migration is a factor known to influence COVID-19 risk, we targeted this group in the analysis by including migration background as an independent variable in our analyses to describe factors associated with impaired HRQoL [14, 15]. Applying the OECD equivalence scale, we estimated the adjusted household income utilizing the variables of net household income, household size, number of children per household, and income statistics for Berlin, Germany [16, 17].

To assess HRQoL, we used three tools of the Patient-Reported Outcomes Measurement Information System® (PROMIS®): PROMIS-29 Profile (v2.1), PROMIS Short Form v1.0 – Dyspnea Functional Limitations 10a, and PROMIS Short Form v2.0 – Cognitive Function 4a for adults [18–22]. PROMIS-29 comprises eight domains: physical function, anxiety, depression, fatigue, sleep disturbance, ability to participate in social roles and activities (hereafter, ‘social abilities’), pain interference, and pain intensity [23]. These tools almost exclusively assess HRQoL of the seven days prior to completion of the questionnaire [24]. We converted raw data to referenced T-scores using the HealthMeasures Scoring Service (https://www.assessmentcenter.net/ac_scoringservice). In that, T-scores are related to data from a reference population, whose basic demographic characteristics match the 2000 United States (US) census [25]. Missing data were accounted for by response pattern scoring [23, 26, 27]. Raw scores are provided in the supplementary material available from the Cambridge Core website (Supplementary Table S1). The meaning of the T-scores varies with domains: higher T-scores in physical function, social abilities, and cognitive function represent better HRQoL, while higher T-scores in anxiety, depression, fatigue, sleep disturbance, pain interference, and dyspnoea represent poorer HRQoL [25]. The highest raw score converts to a T-score of 57.0–81.6 depending on the domain [23, 26, 27]. In accordance with a PROMIS leadership consensus, we considered a difference between groups of at least three T-score points to reflect a meaningful difference [25]. Next, we dichotomized T-scores into normal *versus* at least mildly impaired (hereafter, ‘impaired HRQoL’) using score cut points [25]. For the purpose of this study, we set T-score thresholds for impaired HRQoL at T-scores of 45 (physical function, social abilities, and cognitive function) or 55 (anxiety, depression, fatigue, and pain interference) [25]. The domain pain intensity is scored on a numeric scale from 0 to 10, and no T-scores are available [23]. The reference populations of the domains sleep disturbance and dyspnoea are not based on the general population [25]. Consequently, T-score thresholds derived from the reference population of these domains are not applicable for our population.

### Data analysis

The paper-based questionnaire was digitized using FormPro Software (OCR Systems, Version 3.1) and transferred into Microsoft Excel 2016. We used SPSS IBM version 28.0.1.0 and R (4.3.0) for statistical analyses. Socio-demographic characteristics are presented as median and range for continuous variables and as proportions for categorical variables. HRQoL scores are presented as means with standard deviation (SD), differences between mean T-scores with corresponding 95% confidence interval (CI) are included. Following dichotomization, impaired HRQoL is reported separately as a categorical variable for each time interval. Due to the explorative aim of this study to describe factors associated with impaired HRQoL, we have included every dichotomized domain in further analyses. Potential associations between the factors tested and HRQoL impairment were evaluated in mixed-effects univariable logistic regression models among former COVID-19 patients. In that, variables to be included were determined by consensus by a subset of the authors. Each potential factor was included as independent variable with each HRQoL domain as dependent variable in individual models. To adjust for the potential clustering of data representing three different time intervals, random intercepts were included in the models; no further adjustments were made in this descriptive study. The reported odds ratios (OR) are used descriptively to summarize observed relationships without implying causation. This study did not include a causal framework.

## Results

We contacted all 2991 individuals who had tested positive for SARS-CoV-2 at the Charité public testing site, of whom 576 responded (response rate, 19.3%). We then contacted 4869 out of the 20700 individuals who had tested negative, of whom 693 responded (response rate, 14.2%). [12] After exclusion of initially non-infected individuals who reported confirmed or highly suspected COVID-19 (n = 391) by the aforementioned definition and minors (n = 21) from SARS-CoV-2 infected and non-infected participants, we included 559 former COVID-19 patients and 298 consistently non-infected individuals in this analysis.

### Socio-demographic characteristics

Socio-demographic characteristics are presented in Table 1. The median time interval between SARS-CoV-2 test date and study participation was 14 months (range, 6–26 months). The median age of participants was 40 years (range, 18–82 years). Almost two thirds of the participants were female. Of all former COVID-19 patients, 6% had formerly been hospitalized due to symptoms of COVID-19. At the time of study participation, 98.6% of all participants had received at least one dose of SARS-CoV-2 vaccine. Among vaccinated former COVID-19 patients, the vast majority (97.8%) had received the first dose after infection. The majority, 69.7%, of participants with migration background were German citizens. Other nationalities included primarily European or North American countries.

**Table 1.** Socio-demographic characteristics of former COVID-19 patients and non-infected individuals at the time of study participation

	SARS-CoV-2 non-infected: all (n = 298)	Former COVID-19 patients			
		All former COVID-19 patients (n = 559)^*^	i1 (≤12 months) (n = 152)	i2 (>12–18 months) (n = 298)	i3 (>18 months) (n = 103)
**Age**					
Years^**†**^	46 (19–80)	39 (18–82)	38 (18–73)	38 (18–82)	43 (22–74)
Missing	10 (3.4)	6 (1.1)	0	0	0
**Sex**					
Female	189 (64.7)	322 (58.0)	102 (67.1)	170 (57.4)	48 (47.1)
Male	102 (34.9)	232 (41.8)	50 (32.9)	125 (42.2)	54 (52.9)
Diverse	1 (0.3)	1 (0.2)	0	1 (0.3)	0
Missing	6 (2.0)	4 (0.7)	0	2 (0.7)	1 (1.0)
**Interval: test to study participation**					
Months^**†**^	15 (9–25)	13 (6–26)	10 (6–12)	14 (13–18)	22 (19–26)
Missing	10 (3.4)	6 (1.1)	0	0	0
**Formal education**					
Lower	26 (8.9)	60 (10.8)	16 (10.7)	28 (9.4)	12 (11.7)
Higher academic	229 (78.7)	418 (75.3)	109 (72.7)	227 (76.4)	81 (78.6)
Higher non-academic	36 (12.4)	77 (13.9)	25 (16.7)	42 (14.1)	10 (9.7)
Missing	7 (2.3)	4 (0.7)	2 (1.3)	1 (0.3)	0
**Status of employment**					
Working	231 (78.6)	431 (77.7)	123 (82.0)	224 (76.7)	79 (77.5)
Job seeking	8 (2.7)	15 (2.7)	6 (4.0)	5 (1.7)	4 (3.9)
In education	20 (6.8)	56 (10.2)	16 (10.7)	38 (13.0)	2 (2.0)
Retired	30 (10.3)	37 (6.7)	2 (1.3)	20 (6.8)	15 (14.7)
Inability to work	3 (1.0)	10 (1.8)	3 (2.0)	5 (1.7)	2 (2.0)
Other	2 (0.7)	6 (1.1)	2 (1.3)	3 (1.9)	1 (1.0)
Missing	4 (1.3)	4 (0.7)	2 (1.3)	6 (2.0)	1 (1.0)
**Household income**					
Above average	82 (37.3)	138 (32.2)	35 (29.2)	77 (33.3)	26 (34.7)
Average	94 (42.7)	180 (42.0)	51 (42.5)	95 (41.1)	32 (42.7)
Below average	44 (20.0)	111 (25.9)	34 (28.3)	59 (25.5)	17 (22.7)
Missing	78 (26.2)	130 (23.3)	32 (21.1)	67 (22.5)	28 (27.2)
**Migration background**					
Yes	62 (21.1)	165 (29.8)	43 (28.7)	90 (30.4)	30 (29.1)
No	232 (78.9)	389 (70.2)	107 (71.3)	206 (69.6)	73 (70.9)
Missing	4 (1.3)	5 (0.9)	2 (1.3)	2 (0.7)	0
**Hospital admission due to COVID–19 symptoms**					
Yes	—	33 (5.9)	6 (3.9)	14 (4.7)	12 (11.7)
No	—	526 (94.1)	146 (96.1)	284 (95.3)	91 (88.3)
Missing	—	0	0	0	0

* Six former COVID-19 patients could not be grouped into i1-i3 due to missing data.

† Presented as median (range); all other variables incl. all missing values are presented as n (%).

### Differences in HRQoL scores between former COVID-19 patients and non-infected individuals

Mean T-scores of former COVID-19 patients and consistently non-infected individuals with SD as well as the difference of these means with 95% CI are presented in Table 2. Throughout the period of observation, T-scores were worse in former COVID-19 patients, with few exceptions. For the shortest time interval (i1, ≤12 months), the threshold of a meaningful difference between former patients and non-infected was exceeded for the domains fatigue, sleep disturbance, cognitive function, and dyspnoea reflecting worse HRQoL in former COVID-19 patients. More than 12 months after COVID-19 (i2 and i3), such was observed only for the domain cognitive function.

**Table 2.** HRQoL: unadjusted mean T-scores in former SARS-CoV-2 non-infected individuals and former COVID-19 patients

	Non-infected/infected: i1 (≤12 months)	Non-infected/infected: i2 (>12−18 months)	Non-infected/infected: i3 (>18 months)
Physical function	**54.5** (5.8)/**51.6** (7.7); 2.9 (1.5 to 4.4)	**54.5** (5.8)/**51.9** (7.0); 2.7 (1.6 to 3.7)	**54.5** (5.8)/**52.4** (7.2); 2.1 (0.6 to 3.7)
Anxiety	**49.6** (8.5)/**50.4** (9.1); −0.8 (−2.6 to 1.0)	**49.6** (8.5)/**50.0** (9.4); −0.4 (−1.9 to 1.1)	**49.6** (8.5)/**50.6** (10.4); −0.9 (−3.2 to 1.3)
Depression	**50.2** (8.7)/**50.8** (9.2); −0.6 (−2.4–1.2)	**50.2** (8.7)/**51.0** (9.1); −0.8 (−2.3 to 0.6)	**50.2** (8.7)/**49.7** (9.3); −0.4 (−1.6 to 2.5)
Fatigue	**48.0** (10.0)/**51.1** (11.2); −3.1 (−5.3 to (−1.0))	**48.0** (10.0)/**50.8** (10.5); −2.8 (−4.5 to (−1.1))	**48.0** (10.0)/**48.4** (11.4); −0.4 (−2.9 to 2.1)
Sleep disturbance	**47.5** (8.3)/**51.0** (8.5); −3.5 (−5.2 to (−1.8))	**47.5** (8.3)/**50.4** (8.8); −2.8 (−4.2 to (−1.4))	**47.5** (8.3)/**48.9** (9.7); −1.4 (−3.5 to 0.8)
Social abilities	**53.6** (8.9)/**52.2** (9.3); 1.4 (−0.4 to 3.3)	**53.6** (8.9)/**51.8** (9.3); 1.8 (0.3 to 3.3)	**53.6** (8.9)/**52.6** (9.9); 1.0 (−1.2 to 3.2)
Pain interference	**47.8** (7.6)/**48.5** (9.0); −0.8 (−2.5 to 1.0)	**47.8** (7.6)/**48.2** (8.2); −0.4 (−1.7 to 0.9)	**47.8** (7.6)/**47.5** (8.3); 0.3 (−1.6 to 2.1)
Pain intensity^†^	**1.4** (1.8)/**1.6** (2.1); −0.2 (−0.6 to 0.2)	**1.4** (1.8)/**1.6** (1.9); −0.2 (−0.5 to 0.2)	**1.4** (1.8)/**1.3** (2.1); 0.1 (−0.4 to 0.5)
Cognitive function	**52.0** (8.4)/**48.6** (10.7); 3.4 (1.4 to 5.4)	**52.0** (8.4)/**48.1** (9.6); 3.9 (2.4 to 5.4)	**52.0** (8.4)/**48.7** (11.7); 3.3 (0.8 to 5.8)
Dyspnoea	**38.1** (6.2)/**41.8** (8.9); −3.7 (−5.3 to (−2.0))	**38.1** (6.2)/**40.3** (7.6); −2.2 (−3.3 to (−1.0))	**38.1** (6.2)/**40.4** (8.7); −2.3 (4.2 to (−0.5))

Data are mean T-Score (standard deviation) of non-infected individuals/mean T-Score (standard deviation) of former COVID-19 patients; difference of T-Score means between SARS-CoV-2 non-infected and former COVID-19 patients (95% confidence interval).

† For the domain pain intensity, no T-score is generated, presented scores are raw scores. T-Scores and raw scores are bolded. Frequency of missing values and usage of response pattern scoring is found in Supplementary Table S1.

**Figure 1.**
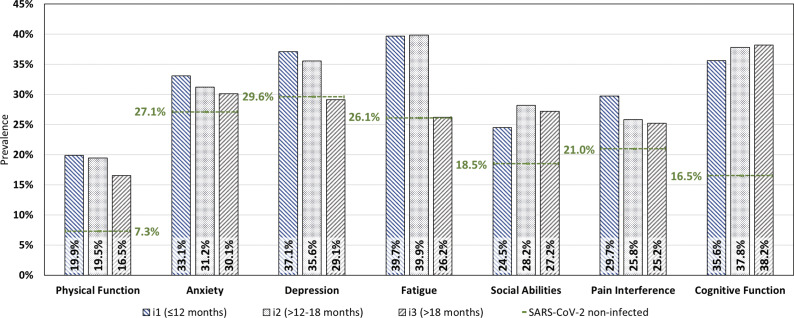
Impaired HRQoL among former COVID-19 patients and non-infected individuals.

Next, we dichotomized T-scores into normal and impaired HRQoL (Figure 1). More than one in four non-infected individuals exhibited impaired HRQoL in the domains anxiety (n = 74, 27.1%), depression (n = 81, 29.6%), and/or fatigue (n = 71, 26.1%). However, impaired HRQoL in these domains was more frequent in former COVID-19 patients. Formerly SARS-CoV-2-infected individuals demonstrated a higher prevalence of impaired HRQoL than non-infected individuals in all domains up to 18 months (i1 and i2) after COVID-19. An impairment in the domains physical (19.2% of all former COVID-19 patients vs. 7.3% in non-infected) and cognitive function (37.6% of all former COVID-19 patients vs. 16.5% in non-infected) was two to three times more frequent in former COVID-19 patients compared to non-infected individuals. In the domain physical function, anxiety, depression, and pain interference at increasing time intervals between test and study participation, the prevalence of impaired HRQoL demonstrated to gradually decrease among former COVID-19 patients.

### Factors associated with impaired HRQoL after COVID-19

Finally, we analysed associations of socio-demographic and socio-economic factors with impaired HRQoL among former COVID-19 patients (Table 3). For an impairment in the domains physical function, anxiety, social abilities, pain interference, and cognitive function, associations were stronger for lower education (OR 5.3/2.5/1.9/4.5/2.0) and COVID-19-related hospitalization (OR 4.2/2.3/3.8/3.0/4.7) than other factors. For the domain social abilities, an equal association as for lower education was found for an income below average (OR 1.9). For the domain depression, positive ORs were found for COVID-19-related hospitalization (OR 2.6) and an income below average (OR 2.5), among others. For fatigue, this was the case for an income below average (OR 2.3) and female sex (OR 2.2). As a factor, migration background demonstrated stronger associations with an impairment in the mental health domains anxiety and depression as compared to other domains.

**Table 3.** Factors associated with impaired HRQoL among former COVID-19 patients

		Physical function	Anxiety	Depression	Fatigue	Social abilities	Pain interference	Cognitive function
Age >40 years		1.9 (1.3–3.0)	1.0 (0.7–1.5)	1.0 (0.7–1.4)	1.0 (0.7–1.4)	1.5 (1.0–2.2)	2.1 (1.5–3.1)	1.3 (0.9–1.9)
Female sex		1.7 (1.1–2.6)	2.2 (1.5–3.2)	1.5 (1.0–2.1)	2.2 (1.5–3.1)	1.4 (0.9–2.0)	2.0 (1.4–3.0)	1.6 (1.1–2.2)
Education	Higher non–academic	3.0 (1.7–5.2)	1.9 (1.1–3.1)	1.5 (0.9–2.4)	1.5 (0.9–2.5)	1.5 (0.9–2.5)	1.9 (1.1–3.2)	1.3 (0.8–2.1)
	Lower	5.3 (2.9–9.6)	2.5 (1.4–4.4)	2.2 (1.2–3.8)	1.3 (0.7–2.3)	1.9 (1.0–3.4)	4.5 (2.6–8.1)	2.0 (1.1–3.4)
Income	Above average	0.8 (0.4–1.5)	1.1 (0.7–1.8)	1.1 (0.7–1.7)	1.2 (0.8–2.0)	0.8 (0.5–1.4)	1.6 (0.9–2.8)	1.2 (0.7–1.9)
	Below average	2.1 (1.2–3.7)	1.8 (1.1–3.0)	2.5 (1.6–4.2)	2.3 (1.4–3.7)	1.9 (1.1–3.1)	2.1 (1.2–3.7)	1.8 (1.1–2.9)
Migration background		1.2 (0.8–2.0)	1.9 (1.3–2.8)	2.2 (1.5–3.2)	1.0 (0.7–1.5)	1.6 (1.0–2.3)	1.4 (0.9–2.1)	1.4 (0.9–2.0)
COVID-19 hospitalization		4.2 (2.0–8.8)	2.3 (1.1–4.7)	2.6 (1.2–5.3)	1.2 (0.6–2.6)	3.8 (1.9–7.9)	3.0 (1.4–6.1)	4.7 (2.1–10.4)
Time interval	i2	1.0 (0.6–1.6)	0.9 (0.6–1.4)	0.9 (0.6–1.4)	1.0 (0.7–1.5)	1.2 (0.8–1.9)	0.8 (0.5–1.3)	1.1 (0.7–1.7)
	i3	0.8 (0.4–1.5)	0.9 (0.5–1.5)	0.7 (0.4–1.2)	0.5 (0.3–0.9)	1.2 (0.7–2.0)	0.8 (0.5–1.4)	1.1 (0.7–1.9)

Data are presented as odds ratio (95% confidence interval).

## Discussion

In this study, we assessed HRQoL among former COVID-19 patients and consistently non-infected participants who had been tested 6 to 26 months prior to study participation. HRQoL T-scores were lower among former COVID-19 patients compared to non-infected individuals across multiple domains. The reduction in HRQoL scores among former COVID-19 patients was more pronounced the more recent the positive test, with one notable exemption, cognitive function. For that, a meaningful difference compared to non-infected individuals was present at all time intervals we assessed. After dichotomization, prevalence of impaired HRQoL was higher among former COVID-19 patients than non-infected individuals in all domains and most time intervals. Various socio-demographic and socio-economic factors, as well as prior hospitalization due to COVID-19, demonstrated to be associated with HRQoL impairments in various domains among former patients.

Across multiple studies, including different populations, periods, and assessment tools, it has been demonstrated that HRQoL scores commonly improve over the months following COVID-19 [28–30]. Our descriptive approach may support this trend. However, we did not follow-up on intra-individual changes of HRQoL and are, therefore, not directly describing recovery from HRQoL impairments. Longitudinal data are needed to show improvements over time.

Mean T-scores in our cohort were within normal limits when utilizing score thresholds [25]. This accords with other studies, which report inferior mean HRQoL scores in former COVID-19 patients than reference population means while formally being largely within normal limits of a US reference population [8, 31]. Most PROMIS reference scores are based on the 2000 US census and can consequently not account for potential shifts due to the COVID-19 pandemic [25]. According to a PROMIS leadership consensus, a meaningful minimal difference of three T-score points ‘may be reasonable for most contexts’ [25]. However, there is no threshold that always reflects a meaningful difference. Therefore, thresholds in general also come with uncertainties, and results should be interpreted cautiously. While numerous studies on post-COVID-19 HRQoL reported only the statistical significance between score differences, it is crucial to consider the clinical context and relevance of such differences, since statistical significance of minor differences can often be reached by sufficiently large sample sizes [32]. Therefore, statistically significant differences of HRQoL scores without clinical context need to be interpreted with caution.

In our cohort, migration background demonstrated associations with impairment in two mental health domains (anxiety and depression). Verveen *et al.* reported from a prospective study among former COVID-19 patients in the Netherlands that migration background was associated with inferior HRQoL in multiple domains [10]. For the mental health domain, this was more pronounced for a migration background from high-income countries [10]. More targeted research is needed to better understand the linkages between COVID-19, migration, and mental health.

It has been demonstrated that vaccination prior to infection lowers the risk for health consequences after COVID-19 [33]. The vast majority of our cohort was vaccinated after SARS-CoV-2 infection. Therefore, we do not assume that vaccination status had a strong influence on our results. It has also been demonstrated that health consequences after SARS-CoV-2 infection were more common in the pre-Omicron era of the pandemic [3]. Before service of our testing site was terminated, SARS-CoV-2 infections were caused by Delta- or previous variants [34]. Because COVID-19 always has and always will be a continuously evolving challenge, our results offer a snapshot of HRQoL data earlier in the pandemic and should be applied cautiously in the current COVID-19 context.

## Strengths and limitations

This study has several strengths. Firstly, while most research on health outcomes after COVID-19 stopped at 6–12 months post-infection, our study recorded HRQoL after a long interval of up to 26 months following the positive SARS-CoV-2 test. While many studies on post-COVID-19 HRQoL focus on specific subgroups, e.g. hospitalized patients or patients suffering from rheumatic diseases, we aimed at addressing the general population [35–37]. Another strength is the use of an established tool for HRQoL assessment allowing for comparison to past and future studies. Finally, findings in former COVID-19 patients could be compared to a reference group of consistently non-infected individuals living under the same pandemic interventions such as lockdowns and social distancing measures.

As to limitations, firstly, due to a highly dynamic epidemic situation, many of the initially negatively tested individuals had meanwhile been infected too. Therefore, the initial goal of a balanced study population of former COVID-19 patients and non-infected individuals was not met. Secondly, questionnaires were available only in a limited number of languages, which might have led to an exclusion bias of certain migrant groups. This is mirrored in the fact that in our study population, individuals with migration background were underrepresented as compared to Berlin reference values [15]. Participation was voluntary. Consequently, the existence of a participation bias cannot be ruled out. Indicators for such bias are the relatively low response rate, the number of vaccinated individuals, and the imbalance of missing values (Supplementary Table S1). Individuals suffering from symptoms after acute COVID-19 might have been more motivated to participate in the present study, which would reduce its generalizability. We investigated individuals with migration background as one group, not considering countries of origin of individuals and/or their parents specifically. The definition by the German Federal Statistical Office could include a diverse group of individuals. However, only a very small proportion of our cohort reported to be citizens of countries from which migration to Germany due to war, oppression, and other exposures presumably resulting in higher rates of impaired HRQoL was common. Consequently, in our case, we do not assume that this relevantly altered the results. Because data collection among former COVID-19 patients and non-infected individuals did not occur simultaneously, it is imaginable that seasonal influences on HRQoL slightly skewed the results, e.g. lower rate of depression among individuals who participated in summer as compared to winter. However, since on median, study participation of non-infected individuals occurred only two months later, we do not assume that a seasonal influence heavily skewed our results. Participants had to survive to participate in our study, excluding potential impact of COVID-19 on HRQoL among those individuals who died. The PROMIS measures used to investigate current HRQoL almost exclusively refer to the last 7 days [24]. Therefore, the assessment could be influenced, e.g. by current illness or comorbidity. Finally, this is a descriptive study. Further research with a suitable causal framework is needed to establish risk factors of impaired HRQoL after COVID-19.

## Conclusion

More than 5 years after the emergence of SARS-CoV-2, a standardized approach to screening and management of HRQoL impairments after COVID-19 has not been established. Our data suggest that a considerable proportion of COVID-19 patients experience HRQoL limitations after one and a half years, and for physical function and cognitive function even longer. Beyond individual suffering, the societal costs of these HRQoL limitations are unknown, but – considering that COVID-19 affects practically the entire population – presumably high. This underscores the urgent need for more in-depth analysis of the true burden of disease from post-COVID-19 HRQoL reductions and impairments, their causes and clusters in specific populations, as well as, most importantly, the need to identify entry points to care and provide access to them.

## Supporting information

Abucar et al. supplementary material Abucar et al. supplementary material

## Data Availability

The data presented are available upon reasonable request from the corresponding author after publication.

## Supplementary material

For supplementary material accompanying this paper visit http://doi.org/10.1017/S0950268825000366. click here to view supplementary material

## References

[1] O’Mahoney LL, et al. (2023) The prevalence and long-term health effects of long Covid among hospitalised and non-hospitalised populations: a systematic review and meta-analysis. eClinicalMedicine 55, 101762. 36474804 10.1016/j.eclinm.2022.101762 PMC9714474

[2] Woodrow M, et al. (2023) Systematic review of the prevalence of long COVID. Open Forum Infectious Diseases 10, ofad233. 37404951 10.1093/ofid/ofad233 PMC10316694

[3] Xie Y, Choi T and Al-Aly Z (2024) Postacute sequelae of SARS-CoV-2 infection in the pre-delta, delta, and omicron eras. New England Journal of Medicine 391, 515–525. 39018527 10.1056/NEJMoa2403211 PMC11687648

[4] Malik P, et al. (2022) Post-acute COVID-19 syndrome (PCS) and health-related quality of life (HRQoL)-A systematic review and meta-analysis. Journal of Medical Virology 94, 253–262. 34463956 10.1002/jmv.27309 PMC8662132

[5] Crispin J (2024) Quality of Life: Encyclopedia Britannica. https://www.britannica.com/topic/quality-of-life (accessed 4 March 2024).

[6] Nandasena H, et al. (2022) Quality of life of COVID 19 patients after discharge: systematic review. PLoS One 17, e0263941. 35171956 10.1371/journal.pone.0263941 PMC8849513

[7] Wachtler B, et al. (2020) Socioeconomic inequalities and COVID-19 – A review of the current international literature. Journal of Health Monitoring 5, 3–17. 10.25646/7059 PMC8734114 35146298

[8] Case KR, et al. (2022) Health-related quality of life and social determinants of health following COVID-19 infection in a predominantly Latino population. Journal of Patient Reported Outcomes 6, 72. 35737279 10.1186/s41687-022-00473-8 PMC9219362

[9] Xiao XY, et al. (2023) Influencing factors associated with quality of life and depression among COVID-19 survivors during convalescence. Psychology, Health & Medicine 28, 2501–2511. 10.1080/13548506.2023.2224037 37314116

[10] Verveen A, et al. (2022) Health-related quality of life among persons with initial mild, moderate, and severe or critical COVID-19 at 1 and 12 months after infection: a prospective cohort study. BMC Medicine 20, 422. 36324167 10.1186/s12916-022-02615-7 PMC9629769

[11] Maechler F, et al. (2020) Epidemiological and clinical characteristics of SARS-CoV-2 infections at a testing site in Berlin, Germany, March and April 2020-a cross-sectional study. Clinical Microbiology and Infection 26(1685), e7–e12. 10.1016/j.cmi.2020.08.017 PMC7438270 32827715

[12] Meierkord A, et al. (2025) Post-infection symptoms up to 24 months after COVID-19: a matched cohort study in Berlin, Germany. Frontiers in Public Health 13, 1513664. 40145003 10.3389/fpubh.2025.1513664 PMC11937017

[13] Statistisches Bundesamt (Destatis). Migrationshintergrund. https://www.destatis.de/DE/Themen/Gesellschaft-Umwelt/Bevoelkerung/Migration-Integration/Glossar/migrationshintergrund.html (accessed 17 May 2024).

[14] Hayward SE, et al. (2021) Clinical outcomes and risk factors for COVID-19 among migrant populations in high-income countries: a systematic review. Journal of Migration and Health 3, 100041. 33903857 10.1016/j.jmh.2021.100041 PMC8061095

[15] Amt für Statistik Berlin-Brandenburg. (2021) Einwohnerregisterstatistik . https://download.statistik-berlin-brandenburg.de/b59486392f2d43ff/b5faad3d13a2/SB_A01-05-00_2021h02_BE.pdf (accessed 17 May 2024).

[16] OECD. (2013) OECD Framework for Statistics on the Distribution of Household Income, Consumption and Wealth.

[17] Amt für Statistik Berlin-Brandenburg. (2023) Primäreinkommen und Verfügbares Einkommen der privaten Haushalte im Land Berlin 1991 bis 2021. https://download.statistik-berlin-brandenburg.de/fa73d809092a8353/73818f481e3d/SB_P01-10-00_2021j01_BE.pdf (accessed 17 May 2024).

[18] Hays RD, et al. (2018) PROMIS®-29 v2.0 profile physical and mental health summary scores. Quality of Life Research 27, 1885–1891. 29569016 10.1007/s11136-018-1842-3 PMC5999556

[19] Choi SW, et al. (2011) Development of a conceptual framework and calibrated item banks to measure patient-reported dyspnea severity and related functional limitations. Value Health 14, 291–306. 21402297 10.1016/j.jval.2010.06.001

[20] Yount SE, et al. (2011) Brief, valid measures of dyspnea and related functional limitations in chronic obstructive pulmonary disease (COPD). Value in Health 14, 307–315. 21402298 10.1016/j.jval.2010.11.009

[21] Irwin DE, et al. (2015) Correlation of PROMIS scales and clinical measures among chronic obstructive pulmonary disease patients with and without exacerbations. Quality of Life Research 24, 999–1009. 25307510 10.1007/s11136-014-0818-1 PMC4369165

[22] Lai J-S, et al. (2014) Self-reported cognitive concerns and abilities: two sides of one coin? Psycho-Oncology 23, 1133–1141. 24700645 10.1002/pon.3522 PMC4185008

[23] Health Measures. (2024) PROMIS Adult Profile Instruments Scoring Manual. https://www.healthmeasures.net/images/PROMIS/manuals/Scoring_Manual_Only/PROMIS_Adult_Profile_Scoring_Manual_16Sept2024.pdf (accessed 10 October 2024).

[24] Cella D, et al. (2019) PROMIS(®) adult health profiles: efficient short-form measures of seven health domains. Value Health 22, 537–544. 31104731 10.1016/j.jval.2019.02.004 PMC7201383

[25] Health Measures. Interpret Scores: PROMIS. https://www.healthmeasures.net/score-and-interpret/interpret-scores/promis (accessed 10 October 2024).

[26] Health Measures. PROMIS Dyspnea Scoring Manual 2021. https://www.healthmeasures.net/images/PROMIS/manuals/Scoring_Manuals_/PROMIS_Dyspnea_Scoring_Manual.pdf (accessed 12 November 2023).

[27] Health Measures. (2022) PROMIS Cognitive Function Scoring Manual. https://www.healthmeasures.net/images/PROMIS/manuals/Scoring_Manual_Only/PROMIS_Cognitive_Function_Scoring_Manual_03June2022.pdf (accessed 12 November 2023).

[28] Shah C, Keerthi BY and Gali JH (2023) An observational study on health-related quality of life and persistent symptoms in COVID-19 patients after hospitalization at a tertiary care centre. Lung India 40, 12–18. 36695253 10.4103/lungindia.lungindia_126_22 PMC9894290

[29] Malesevic S, et al. (2023) Physical health-related quality of life improves over time in post-COVID-19 patients: an exploratory prospective study. Journal of Clinical Medicine 12, 4077. 37373770 10.3390/jcm12124077 PMC10298963

[30] Deesomchok A, et al. (2023) Long-term impacts of COVID-19 pneumonia on quality of life: a single institutional pilot study. Healthcare (Basel) 11, 1963. 37444797 10.3390/healthcare11131963 PMC10341595

[31] Li J, et al. (2024) Examining the trajectory of health-related quality of life among coronavirus disease patients. Journal of General Internal Medicine 39, 1820–7. 38169022 10.1007/s11606-023-08575-9 PMC11282031

[32] Rohmann J. (2018) Don’t Confuse “statistical significance” with “importance”. https://s4be.cochrane.org/blog/2018/05/01/dont-confuse-statistical-significance-with-importance/ (accessed 20 March 2025).

[33] Al-Aly Z, Bowe B and Xie Y. (2022) Long COVID after breakthrough SARS-CoV-2 infection. Nature Medicine 28, 1461–1467. 10.1038/s41591-022-01840-0 PMC9307472 35614233

[34] Robert Koch Institut. (2021) Bericht zu Virusvarianten von SARS-CoV-2 in Deutschland. https://www.rki.de/DE/Content/InfAZ/N/Neuartiges_Coronavirus/DESH/Bericht_VOC_2021-06-23.pdf?__blob=publicationFile (accessed 11 October 2024).

[35] Álvarez-Hernández J, et al. (2023) Long-term outcomes in critically ill patients who survived COVID-19: the NUTRICOVID observational cohort study. Clinical Nutrition 42, 2029–2035. 37659250 10.1016/j.clnu.2023.08.008

[36] Halvorsen P, et al. (2023) Health-related quality of life after surviving intensive care for COVID-19: a prospective multicenter cohort study. Scientific Reports 13, 18035. 37865685 10.1038/s41598-023-45346-2 PMC10590404

[37] Hassen LM, et al. (2022) Functional and psychosocial impact of COVID-19 pandemic on rheumatic patients’ quality of life in Saudi Arabia. Quality of Life Research 31, 3229–3239. 35857205 10.1007/s11136-022-03184-1 PMC9297668

