# Supplementary material for: Health-related quality of life up to 2 years after SARS-CoV-2 infection: a descriptive cohort study
Source: Epidemiol Infect. 2025 Mar 31;153:e60. doi: 10.1017/S0950268825000366 (PMC12001138; doi:10.1017/S0950268825000366)
Supplement: Abucar et al. supplementary material [file S0950268825000366sup001.docx]

Supplementary Material

Health-related quality of life up to 2 years after SARS-CoV-2 infection: a descriptive cohort study

Elias A. M. Abucar^1^*, Mascha Kern^2^, Tobias Kurth^2^, Anne Meierkord^1, 3^, Joachim Seybold^4^, Stefanie Theuring^1^, Frank P. Mockenhaupt^1^

1 Institute of International Health, Charité Center for Global Health, Charité – Universitätsmedizin Berlin, Corporate Member of Freie Universität Berlin and Humboldt-Universität zu Berlin, Berlin, Germany

2 Institute of Public Health, Charité – Universitätsmedizin Berlin, Corporate Member of Freie Universität Berlin and Humboldt-Universität zu Berlin, Berlin, Germany

3 Centre for International Health Protection, Robert Koch Institute, Berlin, Germany

4 Medical Directorate, Charité – Universitätsmedizin Berlin, Corporate Member of Freie Universität Berlin and Humboldt-Universität zu Berlin, Berlin, Germany

* Corresponding author: Elias A. M. Abucar, elias.abucar@charite.de

| **Table S1. Unadjusted mean raw scores as well as the frequency of missing data and usage of response pattern scoring of former COVID-19 patients and non-infected individuals** | | | | | |
| --- | --- | --- | --- | --- | --- |
|  | | **SARS-CoV-2 non-infected  (n=298*)** | **Former COVID-19 patients** | | |
|  |  |  | **i1 (≤12 months)  (n=152*)** | **i2 (>12-18 months) (n=298*)** | **i3 (>18 months)  (n=103*)** |
| **Physical Function: mean (SD)** | | 19.4 (1.9) | 18.5 (2.9) | 18.8 (2.3) | 18.9 (2.4) |
|  | Missing observations | 23 (7.7) | 1 (0.7) | 0 | 0 |
|  | RPS applied | 3 (1.1) | 0 | 4 (1.3) | 0 |
| **Anxiety: mean (SD)** | | 6.5 (2.9) | 6.8 (3.2) | 6.8 (3.3) | 6.8 (3.5) |
|  | Missing observations | 25 (8.4) | 1 (0.7) | 3 (1.0) | 0 |
|  | RPS applied | 2 (0.7) | 2 (1.3) | 0 | 3 (2.9) |
| **Depression: mean (SD)** | | 6.6 (3.2) | 7.1 (3.5) | 7.1 (3.6) | 6.6 (3.6) |
|  | Missing observations | 24 (8.1) | 1 (0.7) | 0 | 0 |
|  | RPS applied | 7 (2.6) | 0 | 3 (1.0) | 0 |
| **Fatigue: mean (SD)** | | 8.4 (3.8) | 9.7 (4.6) | 9.7 (4.2) | 8.7 (4.4) |
|  | Missing observations | 26 (8.7) | 1 (0.7) | 2 (0.7) | 0 |
|  | RPS applied | 4 (1.5) | 1 (0.7) | 6 (2.0) | 0 |
| **Sleep Disturbance: mean (SD)** | | 8.9 (3.5) | 10.4 (3.8) | 10.2 (3.9) | 9.4 (4.1) |
|  | Missing observations | 26 (8.7) | 1 (0.7) | 0 | 0 |
|  | RPS applied | 5 (1.8) | 6 (4.0) | 13 (4.4) | 10 (9.7) |
| **Social Abilities: mean (SD)** | | 16.1 (3.8) | 15.3 (4.2) | 15.3 (4.1) | 15.7 (4.2) |
|  | Missing observations | 27 (9.1) | 1 (0.7) | 0 | 0 |
|  | RPS applied | 4 (1.5) | 5 (3.3) | 8 (2.7) | 4 (3.9) |
| **Pain Interference: mean (SD)** | | 5.8 (3.0) | 6.4 (3.7) | 6.1 (3.3) | 5.7 (3.1) |
|  | Missing observations | 26 (8.7) | 4 (2.6) | 0 | 0 |
|  | RPS applied | 2 (0.7) | 0 | 5 (1.7) | 1 (1.0) |
| **Pain Intensity: mean (SD)** | | 1.4 (1.8) | 1.6 (2.1) | 1.6 (1.9) | 1.3 (2.1) |
|  | Missing observations | 26 (8.7) | 4 (2.6) | 0 | 0 |
|  | RPS applied | 0 | 0 | 0 | 0 |
| **Cognitive Function: mean (SD)** | | 17.2 (3.4) | 15.3 (5.0) | 15.3 (4.4) | 15.4 (5.2) |
|  | Missing observations | 25 (8.4) | 6 (3.9) | 7 (2.3) | 1 (1.0) |
|  | RPS applied | 2 (0.7) | 0 | 1 (0.3) | 0 |
| **Dyspnoea: mean (SD)** | | 1.0 (2.3) | 2.7 (4.6) | 1.7 (3.3) | 2.3 (4.6) |
| Missing observations | | 34 (11.4) | 3 (2.0) | 4 (1.3) | 2 (1.9) |
|  | RPS applied | 37 (14.0) | 19 (12.6) | 38 (12.9) | 6 (5.9) |
| If not indicated otherwise data are presented as n (%). RPS: response pattern scoring  * n are the number of observations before exclusion of missing observations or data that was generated with the use of RPS | | | | | |

| **Table S2. Impaired HRQoL in former COVID-19 patients and SARS-CoV-2 non-infected individuals** | | | | |
| --- | --- | --- | --- | --- |
|  | **SARS-CoV-2 non-infected** | **Former COVID-19 patients** | | |
|  |  | **i1 (≤12 months)** | **i2 (>12-18 months)** | **i3 (>18 months)** |
|  | n (%) | n (%) | n (%) | n (%) |
| **Physical Function** | 20 (7.3) | 30 (19.9) | 58 (19.5) | 17 (16.5) |
| **Anxiety** | 74 (27.1) | 50 (33.1) | 93 (31.2) | 31 (30.1) |
| **Depression** | 81 (29.6) | 56 (37.1) | 106 (35.6) | 30 (29.1) |
| **Fatigue** | 71 (26.1) | 60 (39.7) | 118 (39.9) | 27 (26.2) |
| **Social Abilities** | 50 (18.5) | 37 (24.5) | 84 (28.2) | 28 (27.2) |
| **Pain Interference** | 57 (21.0) | 44 (29.7) | 77 (25.8) | 26 (25.2) |
| **Cognitive Function** | 45 (16.5) | 52 (35.6) | 110 (37.8) | 39 (38.2) |
